# The Links between Food Components, Dietary Habits and Gut Microbiota Composition

**DOI:** 10.3390/foods12203780

**Published:** 2023-10-14

**Authors:** Jichun Zhao, Nan Zhao

**Affiliations:** 1 College of Food Science, Southwest University, Chongqing 400715, China; 2 Institute of Agro-Products Processing Science and Technology, Sichuan Academy of Agricultural Sciences, Chengdu 610066, China; demonzn1115@163.com

## 1. Introduction

Hippocrates, the father of medicine, claimed that “All disease begins in the gut”, while modern scientific studies show that a lot of diseases are at least correlated to the gut, which contains trillions of microbes. The Chinese have a famous saying: “Disease enters by the mouth”, which indicates that diets have a key impact on host health. If these two proverbs are combined, they will uncover the well-known fact that food components have an impact on host health that is correlated to the gut microbiota. A complex correlation has been observed between diets and gut microbes. However, interactions between food components and the gut microbiota are still largely unclear, and require further investigation.

## 2. Metabolism of Food Components by Gut Microbiota

It is well-known that food components provide energy for not only the host, but also for the microbes inhabiting the human gut. The gut microbiota contributes to the human metabolism by contributing microbial enzymes for the degradation of polysaccharides and polyphenols, as well as for the synthesis of vitamins. For example, dietary fibers are difficult to digest in the upper gastrointestinal tract, and tend to reach the large intestine. The bacteria in the large intestine mainly degrade dietary fiber into short-chain fatty acids and gases [1]. Phenolic compounds are one of the most important health-promoting substances in plant foods. However, due to their structural complexity and polymerization, polyphenols exert a poor bioavailability and easily accumulate in the lower gastrointestinal tract. The conversion of the phenol buddleoside by human feces bacteria involves several reactions, including deglycosylation, acetylation, methylation and hydroxylation [2]. The human gut microflora transform anthocyanins into gallic, syringic and p-coumaric acids. On the other hand, anthocyanins and their metabolites can significantly improve the growth of gut bacteria, including *Bifidobacterium*, *Lactobacillus* and *Enterococcus* [3]. It is noteworthy that the metabolism of food components usually results from the coaction of the host and the microbiota.

The characterization of gut microbial metabolites in feces or gastrointestinal contents can be performed through untargeted or targeted metabolomics using mass spectrometry (MS) and nuclear magnetic resonance (NMR) spectroscopy. The application of metabolomics with high-throughput DNA and RNA sequencing techniques can build a link between the gut bacteria and food component metabolites.

## 3. Modulation of Gut Microbiota by Food Components

There is accumulating evidence that demonstrates that the gut microbiota is easily shaped by several factors, such as food components, dietary habits, lifestyle, stress and diseases. Nielsen et al. found that lacto-fermented sauerkraut supplements can alleviate irritable bowel syndrome (IBS) symptoms, causing significant gut microbiota compositional changes, which were attributed to the prebiotics in the sauerkraut [4]. Branched-chain hydroxy acids (BCHAs) that are derived via the fermentation of yogurt have been shown to preserve glucose homeostasis and insulin sensitivity in mice with type 2 diabetes, which is partly associated with the gut microbiota [5]. Although interactions between most food components and the gut microbiota have been demonstrated, they are not fully understood.

## 4. Effects of Dietary Habits on the Gut Microbiota

The effects of different dietary habits on the commensal bacterial composition and immune cells have been discussed in detail in the review by Rinninella et al. [6]. Western diets, characterized by high fat and high sucrose, influence the gut microbial composition, causing low microbial diversity and decreases in beneficial *Bifidobacteria* and *Lactobacillus*, as well as affecting the *Firmicutes*/*Bacteroidetes* ratio. Berberine intake has been shown to alleviate gut barrier disruption and inhibit colon inflammation in high-fat-diet-fed mice. Meanwhile, berberine can ameliorate gut microbial dysbiosis and can induce increases in beneficial gut microbiota, including Akkermansia and Parabacteroides [7].

This Special Issue aims to publish high-quality articles on the connections between food components and the gut microbiota, and how diets can modulate gastrointestinal flora compositions to influence host health.
